# The Polycomb Protein and E3 Ubiquitin Ligase Ring1B Harbors an IRES in its Highly Conserved 5′ UTR

**DOI:** 10.1371/journal.pone.0002322

**Published:** 2008-06-04

**Authors:** Erwin Boutsma, Sonja Noback, Maarten van Lohuizen

**Affiliations:** Division of Molecular Genetics, Netherlands Cancer Institute, Amsterdam, The Netherlands; J David Gladstone Institutes, University of California San Francisco, United States of America

## Abstract

Ring1B is an essential member of the highly conserved Polycomb group proteins, which orchestrate developmental processes, cell growth and stem cell fate by modifying local chromatin structure. Ring1B was found to be the E3 ligase that monoubiquitinates histone H2A, which adds a new level of chromatin modification to Polycomb group proteins. Here we report that Ring1B belongs to the exclusive group of proteins that for their translation depend on a stable 5′ UTR sequence in their mRNA known as an Internal Ribosome Entry Site (IRES). In cell transfection assays the Ring1B IRES confers significantly higher expression levels of Ring1B than a Ring1B cDNA without the IRES. Also, dual luciferase assays show strong activity of the Ring1B IRES. Although our findings indicate Ring1B can be translated under conditions where cap-dependent translation is impaired, we found the Ring1B IRES to be cap-dependent. This raises the possibility that translational control of Ring1B is a multi-layered process and that translation of Ring1B needs to be maintained under varying conditions, which is in line with its essential role as an E3 ligase for monoubiquitination of histone H2A in the PRC1 Polycomb protein complex.

## Introduction

The E3 ubiquitin ligase Ring1B is a member of the highly conserved Polycomb group (PcG) protein family, which act as multimeric chromatin modifying complexes. Recent genome-wide analyses by our group and others clearly demonstrated that PcG protein complexes act on large areas of the genome, thereby maintaining selectivity to genes related to growth and development [1]–[6]. Importantly, PcG protein complexes occupy genes involved in initiation of differentiation in embryonic stem cells [4], and co-occupy a significant subset of genes with embryonic stem cell regulators Nanog, SOX2 and Oct4 [3]. Recently, it was demonstrated that upon induction of differentiation by GATA6, Ring1B target genes in mouse ES cells are derepressed and Ring1B localization to target loci is decreased [6]. These observations are consistent with our own observations, showing that genetic inactivation of PcG proteins in mouse ES cells facilitated a differentiation-prone phenotype (Van der Stoop and Boutsma, submitted).

Also, PcG proteins have been suggested to be involved in X-chromosome silencing in female cells. Initial reports suggested that the PcG proteins Eed and EZH2 are involved in initiation of X-chromosome inactivation since they are enriched on the inactive X-chromosome during early stages of inactivation [7]–[10]. Recently however, it was shown that mouse embryos deficient for the PcG protein Eed are capable of initiating and maintaining X-chromosome inactivation [11], [12]. Instead, it was proposed that PcG proteins might protect the inactive X-chromosome from reactivation as a result of differentiation [13].

Composition of PcG complexes varies among cell types and in time, but in general one can distinguish at least two biochemically distinct complexes. Polycomb Repressive Complex 2 (PRC2), also termed the initiation complex, harbors Eed, EZH1 and EZH2 [14], [15]. The other complex is also termed maintenance complex or PRC1, and consists of Ring1B/Rnf2, Bmi1, M33, MPh1, RYBP and homologues, but also transcription factors such as YY1 and E2F6 [16], [17]. Recently, several enzymatic functions have been linked to Polycomb complexes, among which are histone deacetylation [18]–[20], histone methylation [21]–[23] and histone ubiquitination [24], [25]. It is thought that extensive modification of histone tails determines local chromatin structure, either by influencing chromatin structure directly or by recruitment of modifying factors, such as PcG proteins. Ring1B was found to be responsible for the long-known monoubiquitination of histone H2A at K116 or K119 [25]. It was proposed that this chromatin mark may be involved in transcriptional repression by Polycomb proteins [25], [26]. Experimental evidence to support this idea came when loss of Ring1B mediated monoubiquitination of H2A was linked to the release of poised RNA polymerase II and subsequent derepression of target genes [27]. This finding may provide an explanation for the mechansism of gene repression by PcG proteins via histone modification.

Ring1B is the only member of PRC1 that seems to be essential for life, since Ring1B knockout mice die during gestation after a gastrulation arrest [28]. This is in contrast to the relatively mild phenotypes of knockout mice of other PRC1 members, like Bmi1 [29], Mel18 [30], M33 [31] and MPh1 [32], which exhibit developmental defects that can in part be related to deregulation of Hox genes. The Ring1B interacting protein Bmi1 also has an established role in stem cells of both the haematopoietic system [33], [34] as well as the CNS [35], [36], linking PcG complexes to stem cell self-renewal [reviewed in 37].

To date, little is known about the regulation of PcG proteins via inhibiting factors, stimulating factors or on transcriptional, translational or post-translational levels. Nonetheless, it is known that hierarchical recruitment of PcG members is crucial in many situations, suggesting there must be extensive regulation of PcG protein levels. Especially data on post-transcriptional regulation of PcG protein levels is limited, though our previous work revealed the cell-cycle dependent phosphorylation of Bmi1, which we found to correlate with its dissociation from chromatin [38]. Also, we recently showed Bmi1 can be mono-ubiquitinated, as a part of the E3 ubiquitin ligase complex consisting of Spop and Cullin3 that together ubiquitinate variant histone MacroH2A [10]. Monoubiquitination of PcG members could have a signaling function, for example related to its localization, providing a post-translational level of regulation. Here, we report a novel mechanism through which Ring1B is regulated. We identified a functional IRES in the 5′ UTR of Ring1B and demonstrate strong activity of the IRES in several experimental settings, which indicates Ring1B protein levels are (also) regulated by this alternative translation initiation mode.

IRESes represent highly structured 5′ UTR sequences capable of recruiting the translational machinery independent of a cap site. IRES-dependent translation is relatively rare; the vast majority of all eukaryotic proteins are translated via the well-characterized mechanism of cap-dependent translation. This mechanism is dependent on binding of the cap-binding subunit eIF4E of the eIF4F initiation complex to the 5′ m^7^guanosine cap of the mature RNA transcript. The mechanism of recruitment of the initiation complex by an IRES is largely unknown, although several cellular proteins, termed ITAFs for IRES TransActing Factors (i.e. PTB, hnRNP A1 and several initiation factors), have been found to interact with IRESes [39]–[41]. Most IRES-dependent translation does not need functional eIF4E or the eIF4E-binding domain of eIF4G, which are the main targets for inhibition of cap-dependent translation. Taking advantage of this, several conditions are now known which favor IRES-dependent translation over cap-dependent translation. For example, oxidative stress induces the IRES activity of VEGF [42] and Hif1α [43], amino acid starvation induces IRES-mediated translation of cat-1 [44], and the c-myc IRES is specifically active during apoptosis [45], genotoxic stress [46] as well as G_2_M transition [47]. Infection with certain viruses, such as picornaviruses, can also result in impaired translational activity of the host cell. This is a result of cleavage of the cap-binding domain of eIF4G by viral proteases 2A and 3B [48], [49]. Since viral proteins themselves are often translated via IRESes, expression of these proteases favors viral protein translation and dramatically decreases translational activity of the host cell. Viral IRESes are widely used as tools in molecular and cellular biology, providing a means to translate two or more different proteins from a single transcript.

The here described finding that Ring1B harbors a highly active IRES in its 5′ UTR is, to our knowledge, the first time that this alternative translational mechanism is implicated in the regulation of Polycomb group proteins. This adds a new mechanism by which PcG protein levels are regulated in the cell.

## Materials and Methods

### Plasmid construction

The pLL, pLML, pLVL, pLPL and pLEL vectors were a kind gift from Orna Elroy-Stein and have been described previously [50]. pIND-2A was a kind gift from Chaim Kahana and has also been described earlier [51]. The Ring1B IRES was obtained by PCR from Image Clone BG076521, using primers mRing1B-BglII-IRES-fw (5-gaagatctagtctctcatgaatattgagcg-3) and mRing1B-exon1-rev (5-gggctggggcaggagccgaa-3). This fragment was cloned blunt in the EcoRV site in between both Luciferase coding sequences of pLL, resulting in pLRL. A 5′ part of the Ring1B cDNA, but 3′ of the IRES, was then obtained by PCR using primers mRing1B-exon2-fw (5-gggctggggcaggagccgaaatgtctcaggctgtgcagac-3) and mRing1B-ApaI-rev (5-gaagcgggcccgagtaaca-3). This fragment was combined with the PCRed IRES fragment and because of their 20 base pair overlap, a new fragment was obtained by PCR containing the first four exons, including the IRES in the first exon. This fragment was cloned into Ring1B expression constructs (see below) using BglII and ApaI, resulting in expression constructs harboring the Ring1B IRES. pcDNA3.1 (Invitrogen) and MSCVPuro (Clontech) based Ring1B expression constructs were made by cutting both vectors with BglII and XhoI and cloning in a Ring1B cDNA with a BglII site immediately upstream of the translational start and an XhoI site immediately downstream of the translational stop. A C-terminal flag tag was introduced in both constructs by ligation of an NcoI-HpaI PCR-fragment (obtained with primers Ring1B-NcoI-fw (5-gaacaaacccatggaactttattatg-3) and Ring1B-flag-stop-XhE-rev (5-cacccaccaaggagcacaaagactacaaagacgatgacgacaagtgactcgagggaattcc-3).

### 5′RLM-RACE

Amplification and identification of 5′ cDNA ends was done following the manufacturer's instructions (Ambion). The fragments were amplified by PCR using 5′ adapter primers provided by the manufacturer and a 3′ Ring1B outer primer (5-ggtctggccttagtgatctt-3) and subsequently a 3′ Ring1B inner primer (5-tctttgttgccacttctaagg-3). The extension time of the nested PCR was increased to 2 minutes and the annealing temperature was increased to 62 degrees. Fragments were separated on 2% agarose gels and ligated into the TA-vector following the manufacturer's instructions (Invitrogen).

### Cell culture

Cells were cultured in DMEM (GibcoBRL), supplemented with penicillin/streptomycin (GibcoBRL) and 10% FBS (Hyclone). Transfections were done with the calcium phosphate method in 60 cm^2^ dishes with 25 µg plasmid DNA at 60% confluency. 50 ng of pEGFP was co-transfected to verify by fluorescent microscopy whether transfection was efficient and similar between samples within the same experiment. Infections were done with fresh undiluted supernatant from Phoenix producer cells, taken at 48 hours and 72 hours post-transfection, in the presence of polybreen. Induction of pIND-2A with muristerone-A proved to be unnecessary, since the leakiness of the vector's promoter provided enough 2A protein for our assays. 5 µg pIND-2A was cotransfected with the dual luciferase constructs.

### Western blot analysis

Cells were collected in Eppendorf tubes, washed with PBS and lysed in RIPA buffer (1% NP40, 1% SDS, 0.5% DOC, 50 mM Tris-HCl pH 8, 2 mM EDTA) supplemented with N-ethylameinide (NEM, Sigma) and protease inhibitor cocktail (Roche). After measuring concentrations using the Bradford method, equal amounts of protein were loaded onto precast gradient gels (NuPage, Invitrogen) and subjected to SDS-PAGE. Samples were then transferred to nitrocellulose membranes (Schleicher & Schuel). The membranes were blocked for 1 hour with casein blocking buffer (Roche) which was 1∶10 diluted in PBS, and incubated with primary antibodies for 1 hour. After three washes in PBS-Tween (0.5%), secondary antibodies were applied (Zymed goat anti-mouse 1∶20000 diluted, BioSource goat anti-rabbit 1∶10000 diluted) for 30 minutes. After three washes with PBS supplemented with 0.5% Tween-20, the membranes were incubated with ECL Western Blotting detection reagent (Amersham) and exposed to films (Kodak).

### Luciferase assay

48 hours after transfection cells were harvested, lysed and incubated with luciferase substrates as described in the protocol of the Dual Luciferase kit (Promega). Readout of luciferase activity was done with a luminometer (Turner Designs TD-20/20) for three seconds. Lysates were generally diluted ten to a hundred times before subjecting to the assay, to not exceed the measurable range of the luminometer. If undiluted samples had less than one hundred counts, they were considered to be insignificant. Every experiment was at least done three times and with two significant samples per experiment.

### RNA isolation and quantitative real-time PCR (qPCR)

Total RNA was extracted using TRIzol reagent (Invitrogen), followed by DNase treatment and first strand cDNA synthesis using Superscript II reverse transcriptase (Invitrogen). qPCR was performed on 50 ng cDNA on an ABI Prism 7000 using SYBR Green PCR mastermix (Applied Biosystems). We used two different primer sets: Ring1B-1-fw (5-aaatgtctcaggctgtgcag-3) and Ring1B-1-rev (5-tttccaagccatctgttattgcc-3), and Ring1B-2-fw (5-tcggttttgcgcggatt-3) and Ring1B-2-rev (5-agttttttccgacaggtaggacact-3). Ring1B values were normalized against both Hprt and beta-actin, resulting in the same relative levels.

### Software

Screening of putative IRESes in DNA sequences was done with the web-based software of UTRscan (http://bighost.area.ba.cnr.it/BIG/UTRScan) [52]. Structure prediction of RNA sequences was done with RNAdraw, version 1.1 [53].

## Results

### The Ring1B IRES is highly conserved between mammals

**Figure 1:**
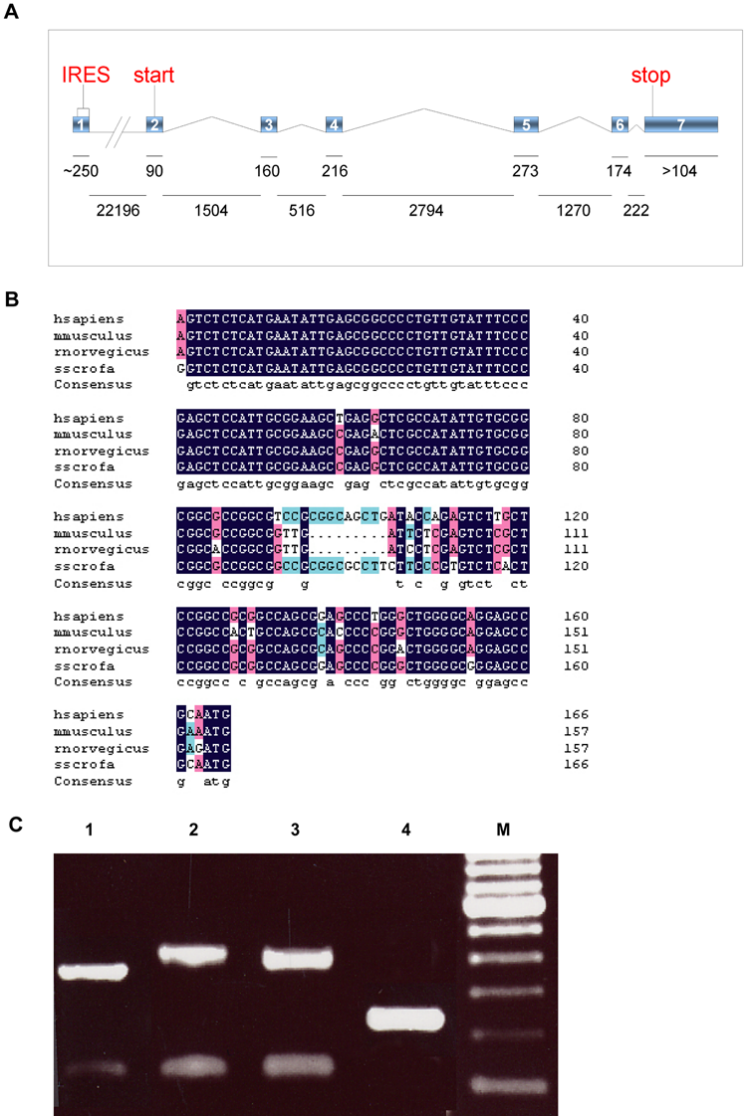
Ring1B has a remarkable genomic structure. The genomic structure of Ring1B is depicted in the upper pane, the approximate sizes of exons in the middle pane and introns in the lower pane, as well as the translational start and stop codons. Exon 7 greatly varies in length, sometimes measuring up to 3,5 kb (A). Apart from a 12 nucleotide stretch in the middle, which is only seen in human and pig Ring1B, IRESes of different mammals are virtually identical. The exact mouse sequence depicted, comprising the entire IRES until the translational start, is used in all our assays (B). A 5′ RLM-RACE was performed to determine the main transcriptional start (C). In lane 5 a 100 bp marker was loaded, with the most prominent band being 600 bp. In ES cells (lane 1), primary MEFs (lane 2) and 293 cells (lane 3) the most abundant fragment is approximately 380 bp long. This suggests a transcriptional start at approximately 154 bp 5′ of the 3′ splice site of the first exon, which was verified after sequencing the fragments. Lane 4 shows an internal control PCR, using the same 3′ Ring1B inner primer, but with a Ring1B 5′ primer instead of an adapter primer. The Ring1B 5′ primer is located downstream of the translational start, so this PCR serves as an internal control for RNA quality and primer specificity.

The *Ring1B* gene is conserved throughout evolution and homologues can be found in mammals, but also for example fruit fly (*Drosohila melanogaster*), zebra fish (*Danio rerio*) and frog (*Xenopus tropicanus*). The mammalian *Ring1B* gene has an unusual structure, which has not been observed in non-mammals. In mice, the first exon is followed by a 22 kb intron, leaving approximately 7.5 kb for the 6 remaining exons (figure 1A). The ORF starts in the second exon and is 1011 bp long, resulting in a 336 aa peptide which can be visualized as a 42 kDa protein on Western blots. Ring1B is ubiquitously expressed *in vivo* in the mouse embryo [54] and highly expressed in a wide range of human, rat and mouse cell types tested, both primary cells and tumor cell lines (data not shown). The genomic sequence in which the first exon is located is very GC-rich, with >80% GC content stretching over several kbs, indicative of a CpG island acting as a promoter. This first exon is very highly conserved between mammalian species, which is unusual for a noncoding sequence (figure 1B).

ESTs in the online databases seem to indicate there are different transcription start sites in this area, sometimes resulting in a transcript with a large 5′ UTR and sometimes in a transcript with a much smaller one. To determine the predominant transcription start site we performed a 5′ RLM-RACE on capped RNA from mouse embryonic stem cells (ES), primary mouse embryonic fibroblasts (MEF) and 293 cells. We found that the majority of the transcripts in all three cell types start at position -154 of the known 3′ splice site of the first exon (figure 1C). Since the translational start is located in the second exon, 2 nucleotides downstream of the 5′ splice site of the second exon, this results in a 5′ UTR of 156 bp. The 5′ UTR has a 70.2% GC content and harbors an out-of-frame alternative start codon. The first in-frame stop codon is found in the second exon, 34 nucleotides downstream of the translational start codon. We cannot rule out that this small ORF plays a regulatory role in Ring1B translation. However, it is unlikely that this ORF is translated efficiently, since it has a very poor Kozak consensus sequence as opposed to the very good Kozak consensus sequence of the Ring1B translational start codon. Moreover, Ring1B constructs lacking the alternative start codon and used in the same experiments described in this paper, gave the same results in our assays (data not shown).

The high degree of sequence conservation suggests there are regulatory sequences located in the Ring1B 5′ UTR. UTRscan, an online source for predicting UTR structures and domains, finds a putative Internal Ribosome Entry Site (IRES) in the 5′ UTR of Ring1B, consisting of 91 nucleotides directly 5′ of the translational start codon.

### The Ring1B 5′ UTR contains a functional IRES sequence

**Figure 2:**
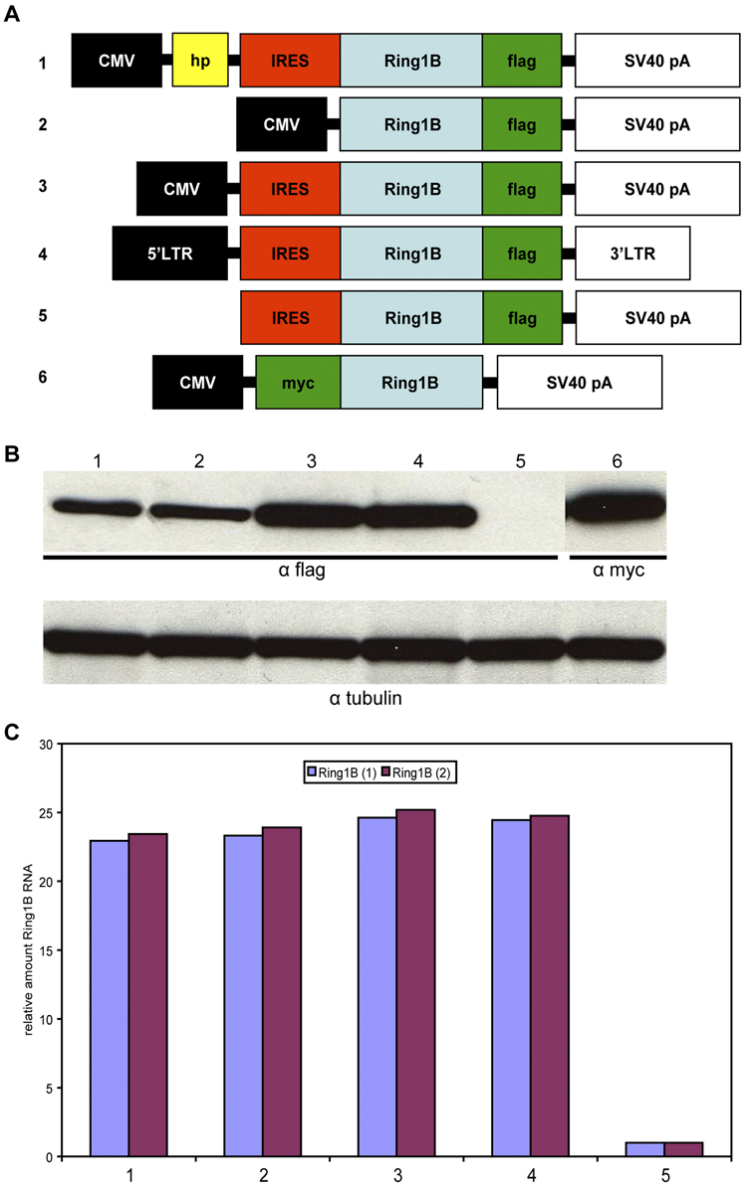
Expression of Ring1B is influenced by the 5′ UTR. In this overview of the Ring1B IRES validation constructs used in our assays, the position of the CMV promoter, the flag and myc tags, the Ring1B ORF, the 140 bp stable hairpin structure (hp) blocking translation, the Ring1B IRES, the SV40 polyadenylation signal and the MSCV-Puro LTRs are depicted (A). Western blots indicate the different expression levels of our Ring1B constructs (B). Numbers correspond to the constructs in figure 2A. All constructs were based on pcDNA3.1 and expression analyses were done with transient transfections in 293 cells, except construct #4, which was based on the retroviral vector MSCV-Puro and was infected in primary MEFs. 50 ng of EGFP-expressing plasmid was cotransfected to be able to judge by fluorescent microscopy whether transfection was comparably efficient. One can clearly appreciate the increased exogenous Ring1B protein levels relative to tubulin when the 5′ UTR is present, as well as the decreased protein levels when the stem-loop-stem hairpin is cloned in front of the 5′ UTR, and the absence of exogenous Ring1B protein when the CMV promoter was removed from the vector. Ring1B RNA levels (both endogenous and transfected measured together) in the same cell populations as in figure 2B were very much alike, ruling out that the differences in protein levels could be accounted for by differences in transcription efficiency (C). RNA levels in this graph were typical for several independent experiments and normalized against beta-actin; normalization against Hprt gave the same result. Ring1B (1) and Ring1B (2) indicate different primer sets. Bar numbers correspond to figure 2A.

We cloned the 154 bp Ring1B 5′ UTR harbouring the putative IRES, based on the results with the RLM-RACE, in front of the Ring1B translational start codon. This resulted in a Ring1B cDNA sequence that also exists as an RNA transcript *in vivo*, apart from the C-terminal flag tag to discriminate it from endogenous Ring1B. As a positive control, we took along N-terminal myc-tagged Ring1B (figure 2A). Expression of Ring1B was readily observed from different vectors, after transient transfection as well as after retroviral infection (figure 2B). Presence of the IRES seemed to increase Ring1B protein levels (figure 2B, lane 3 versus lane 2). Since our construct consist of the entire, most common 5′ UTR, it is slightly larger than the predicted IRES (154 bp versus 91 bp) and harbours the start codon of the small ORF that terminates in the second exon, spanning 186 bp in total.

To rule out a possible effect of this small ORF on translation efficiency, we also tested a construct that consisted of only the Ring1B IRES; there was no significant difference in the results (data not shown). Also, it is unlikely that the differences in Ring1B protein levels are due to differences in transcription efficiency of the plasmids, since we performed quantitative real-time PCR on RNA from these same cells, showing Ring1B RNA levels (both endogenous and transfected were measured together) were very much alike in all transfections (figure 2C).

To further explore the putative IRES function of the Ring1B 5′ UTR, we also made a construct with a small (about 140 bp) synthetic stem-loop-stem structure 3′ of the transcription start site but 5′ of the IRES (figure 2A, construct nr. 1). This hairpin loop, consisting of 8 HindIII restriction sites followed by 10 non-complementary nucleotides and again 8 HindIII restriction sites, is expected to make the cap-dependent 40S scanning subunit of the ribosome dissociate from the transcript, since the eIF4A helicase subunit of the eIF4F translation initiation complex is unable to unwind this highly stable hairpin loop [55]. Thus, in this construct, translation of Ring1B is supposed to be exclusively mediated by non-cap-dependent mechanisms. Indeed we could show Ring1B protein in the presence of the hairpin loop, albeit slightly less than without it (figure 2B, lane 3 versus lane 1). This could be explained by assuming the stem-loop-stem structure causes at least partial disruption of the IRES secondary structure.

### The Ring1B IRES is highly active in many cell lines

**Figure 3:**
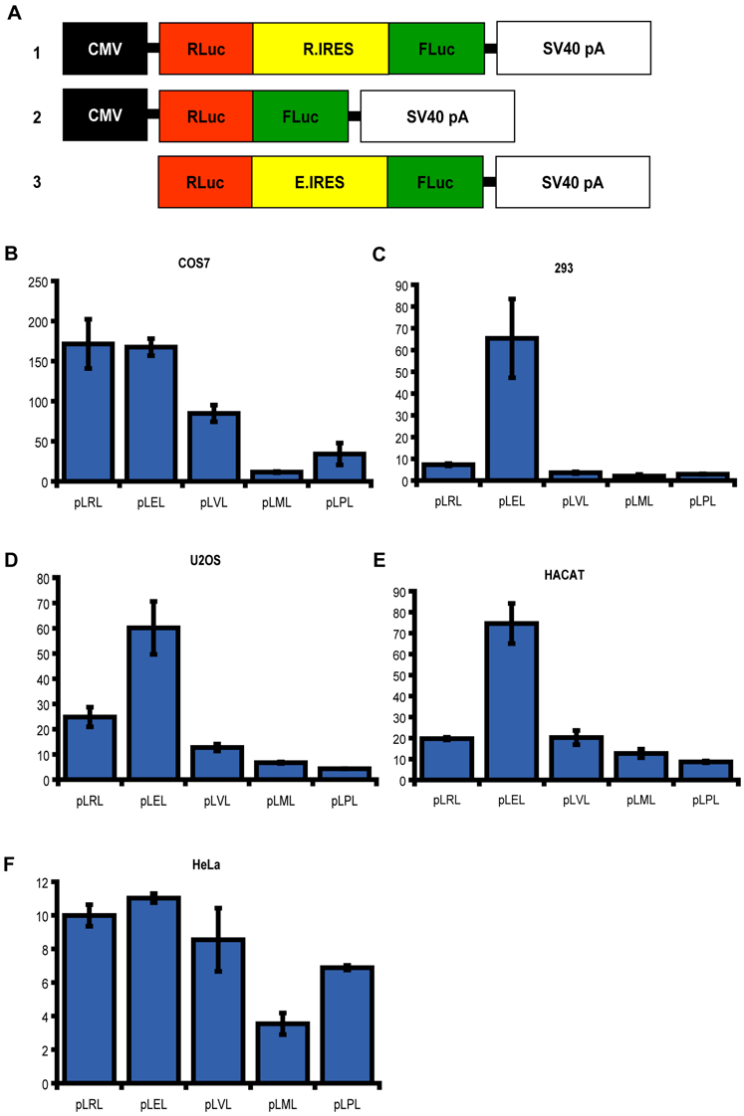
The Ring1B 5′ UTR harbours IRES activity in expression constructs. In this overview of the dual luciferase constructs used in the assays below, the CMV promoter, the Renilla (RLuc) and Firefly (FLuc) luciferase ORFs, the IRESes of EMCV (E.IRES) and Ring1B (R.IRES) and the SV40 polyadenylation signal are depicted (A). The diagrams show activity assays of the cellular IRESes of Ring1B (pLRL), PDGF (pLPL), VEGF (pLVL), c-myc (pLML) and the viral IRES of EMCV (pLEL). The assays were done in COS7 (B), 293 (C), U2OS (d), HACAT (E) and HeLa (F) cells. On the y-axis, the relative activity of the IRES is depicted by dividing the Firefly counts of the sample over the Firefly counts of the empty vector (pLL, which has no IRES between both luciferase ORFs). All samples are corrected for their relative Renilla counts, since Renilla luciferase translation is cap-dependent and generally a readout of transfection efficiency. All experiments were done in duplo and repeated several times. The assays show the varying activity of the IRESes in different cell types. Whereas the EMCV IRES is known to be highly active in most cell types, cellular IRESes are generally much less active. Nevertheless, the Ring1B IRES is as active as the EMCV IRES in COS7 and HeLa cells, and in every cell type the most active IRES among the cellular IRESes.

IRESes have the characteristic feature to be able to recruit the translational machinery independent of its position in a transcript, allowing translation of downstream ORFs. To test the Ring1B IRES in this setting, we cloned it in between a Renilla luciferase ORF and a Firefly luciferase ORF (figure 3A). The resulting construct, pLRL, was expressed from a CMV promoter. We used identical constructs with the cellular IRESes of c-myc (pLML), VEGF (pLVL), PDGF (pLPL) and the viral IRES from the encephalomyocarditis virus (pLEL) as controls. Expression of Renilla is exclusively cap-dependent and is supposed to vary only with varying transfection efficiencies. Therefore, all Firefly readouts were normalized to Renilla luciferase levels. Firefly luciferase activity was the readout of the experiment since it is dependent on the ability of the IRES to recruit the translational machinery. Firefly activity of experimental constructs was divided by the Firefly activity of the construct without IRES (pLL).

In this well-established dual-luciferase assay we compared the activity of the Ring1B IRES by performing transient transfections in COS7, 293, U_2_OS, HACAT and HeLa cells. In all cell types the Ring1B IRES was equally or more active than the control cellular IRESes (figure 3B–F). The EMCV IRES was, with a relative activity between twofold and tenfold over the cellular IRESes, the most active IRES in all but COS7 cells. COS7 cells were the only cell type where a cellular IRES, the Ring1B IRES, was as active as the EMCV IRES. We reproducibly observed that in HeLa cells the EMCV IRES was much less active than in other cell types.

### The Ring1B 5′ UTR does not contain a cryptic promoter

**Figure 4:**
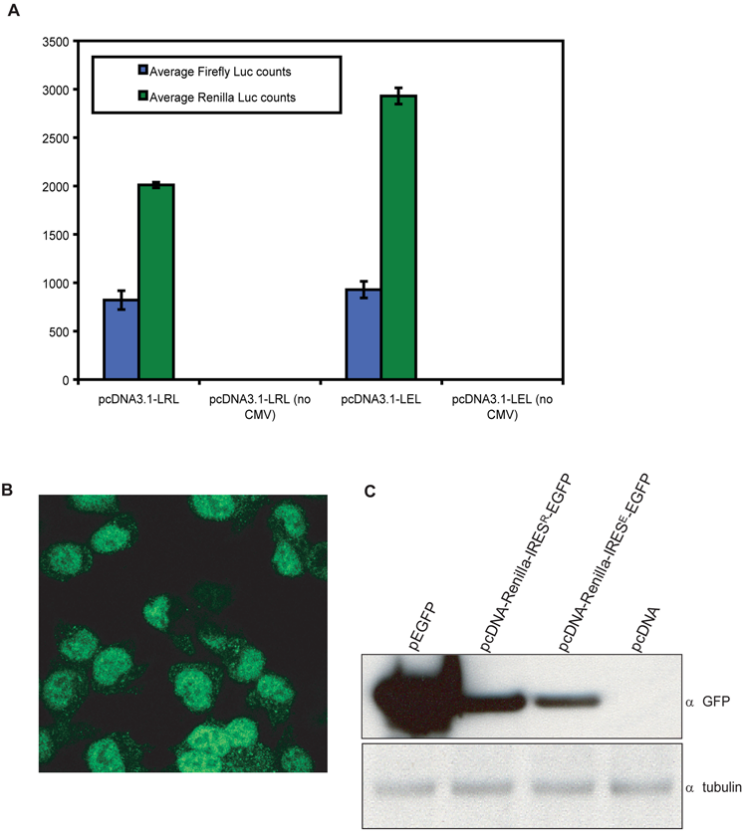
The Ring1B 5′ UTR does not have cryptic promoter activity. To test cryptic promoter activity of the Ring1B 5′ UTR, the Ring1B and EMCV IRES dual luciferase constructs were cloned into a pcDNA3.1 vector without CMV promoter (A). The constructs correspond to the constructs depicted in figure 3A, with Renilla luciferase 5′ of the IRES and Firefly luciferase 3′ of the IRES. The y-axis shows the absolute counts of Firefly luciferase (cap-dependent) and Renilla luciferase (IRES-dependent) after transient transfection in COS7 cells. In the constructs without promoter, no mRNA is produced, therefore no translation occurs and no luciferase activity can be measured, indicating the Ring1B 5′ UTR does not contain a cryptic promoter. pEGFP vector was cotransfected in COS7 cells and showed equal fluorescence in every transfection. In a bicistronic construct with the EGFP ORF downstream of the Ring1B IRES we could clearly detect EGFP fluorescence with fluorescent microscopy (B, Ring1B IRES only) as well as EGFP protein on Western blot (C). The Ring1B IRES (IRES^R^) seems to give a slightly higher expression than the EMCV IRES (IRES^E^), but we did observe some variation in this in our different experiments. On average we would say the EGFP levels were equal. pEGFP served as a positive control, empty pcDNA3.1 as a negative control, and tubulin as a loading control.

Several recent publications state it is hard to discern IRES activity from cryptic promoter activity in GC-rich leader sequences [56], [57]. To exclude this possibility, we cut out the CMV promoter from our Ring1B expression constructs (figure 2A, construct nr. 5) and tested these for Ring1B expression. As expected, no Ring1B protein could be detected on Western blot (figure 2B, lane 5). In addition, we cloned the dual luciferase construct including the Ring1B and, as a control, EMCV IRES in a promoterless pcDNA3.1 construct to test cryptic promoter activity (figure 3A, construct 3). As in our experiment with the promoterless Ring1B construct, which gave no Ring1B expression, the promoterless dual luciferase constructs gave neither Renilla luciferase activity nor Firefly luciferase activity (figure 4A).

Finally, we analyzed the Ring1B IRES and the EMCV IRES in a bicistronic construct with an EGFP coding sequence downstream of the IRES, instead of Firefly luciferase. EGFP fluorescence (figure 4B) as well as EGFP expression (figure 4C) could readily be observed upon transient transfection of the bicistronic constructs, indicating the Ring1B IRES preceding a marker gene could in principle be used as a tool in biological assays to verify transduction efficiency or as a much smaller alternative to viral IRESes.

### Ring1B IRES needs intact eIF4G to function properly

**Figure 5:**
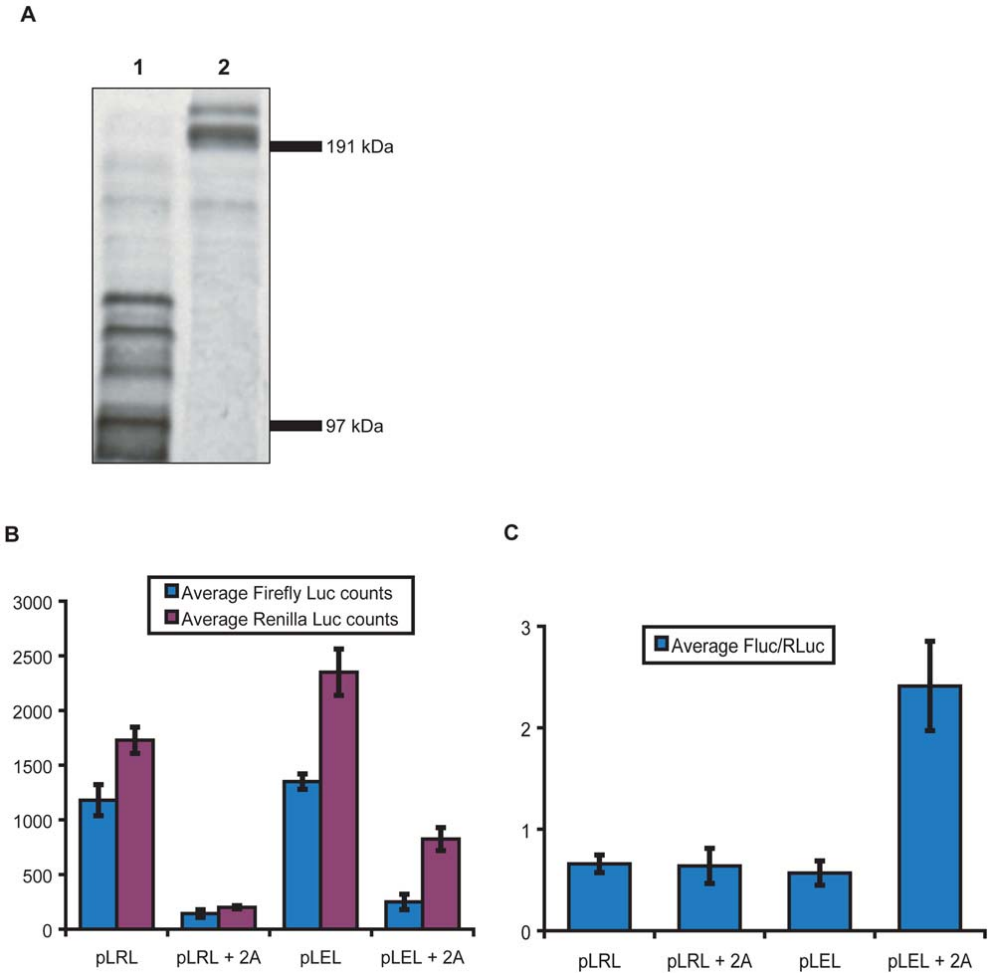
The Ring1B 5′ UTR is sensitive to 2A protease activity. Western blot showing cleaved eIF4GI isoforms upon transfection of viral protease 2A (lane 1) in 293 cells, whereas eIF4GI remains intact in the absence of 2A (lane 2) (A). Molecular weight markers are indicated. This Western blot was done on the same lysates we used in one of the experiments in figure 4B. Results are representative of three independent experiments. The graphs depict the absolute counts of Renilla and Firefly luciferase activity (B) and the ratio of the Firefly luciferase counts over the Renilla luciferase counts (C). Ring1B IRES-dependent Firefly luciferase is clearly affected by the protease to a similar extend as the cap-dependent Renilla luciferase, showing no difference in their relative activity and therefore no difference in the ratio. As a control, in the presence of 2A protease the EMCV IRES-dependent Firefly luciferase is equally active whereas the cap-dependent Renilla luciferase activity is reduced over two-fold, resulting in an over two-fold increase in the ratio. This experiment clearly shows the remarkable apparent cap-dependency of the Ring1B IRES. pIND-2A, the protease-expressing plasmid, was co-transfected at 5 µg per transfection, without muristerone-A induction. Experiments were performed four times. Transfection efficiences were checked to be comparable between samples by cotransfection of 50 ng EGFP-expressing plasmid.

Next, we tested the sensitivity of the Ring1B IRES to viral protease 2A. This protease, isolated from picornaviruses, cleaves off the eIF4E binding domain of eIF4G. Since the binding of eIF4E to the cap site and subsequently to eIF4G is a key step in translation initiation, cap-dependent translation efficiency is greatly reduced in the presence of protease 2A. However, since transcripts harboring an IRES generally do not need a cap site for translation initiation, IRES-dependent translation could still occur. In our assay with the dual luciferase construct, this should result in reduced Renilla luciferase activity but not in reduced Firefly luciferase activity. This is not what we observed upon cotransfection of an inducible protease 2A encoding plasmid, which results in cleaved eIF4G (figure 5A). IRES-dependent Firefly luciferase expression is clearly decreased to a similar extend as cap-dependent Renilla luciferase activity. So whereas the absolute counts dropped, the ratio for both experiments remained equal (figure 5B). The EMCV IRES more than doubles its ratio of IRES-dependent translation over cap-dependent translation with 2A present. This indicates that the Ring1B IRES needs intact eIF4G to function properly.

### Effects of point mutations in the Ring1B IRES correspond to secondary structure prediction

**Figure 6:**
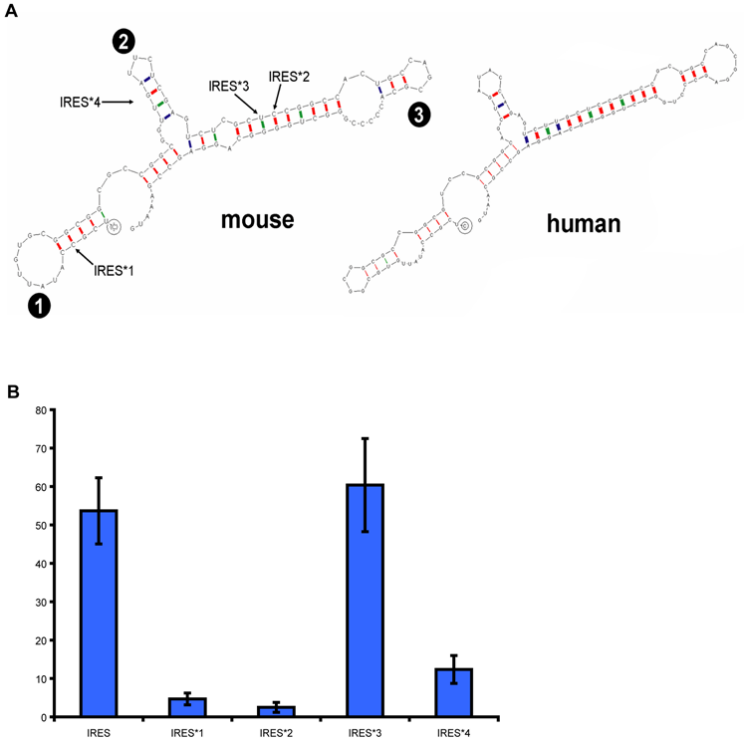
Mouse and human Ring1B 5′ UTRs show a striking structural resemblance. Secondary structure prediction of the Ring1B IRES of human and mouse origin, showing a striking structure similarity (A). The first nucleotide is indicated with a black circle around it, the last triplet is the translational start. Loop numbers are indicated with filled black circles. In mutant IRES*1 and IRES*2 the indicated cytosines are replaced by adenines, creating mismatches. In mutant IRES*3 the indicated uracil is replaced by a cytosine, creating a matching nucleotide and therefore stabilizing the structure. Mutant IRES*4 lacks the entire second loop. Luciferase assays show the remaining activity of the mutant IRESes and the stabilized activity in case of the matching mutation (B). The same construct as in figure 3a, construct number 1, was used, but with the mutations introduced. The way of interpreting the graph is identical to figure 3b–f. The mismatch point mutations almost completely abolish the IRES activity, whereas IRES*3 is, as expected, not affected by its stabilizing point mutation. Surprisingly, the IRES lacking the entire second loop still has some residual activity, indicating the relative importance of the remaining structure.

Next, we analyzed the predicted structure of the 91 bp Ring1B IRES. Although there is no general consensus sequence or structure for IRESes, to know the predicted structure could be of interest to make specific mutants to further validate the IRES or to understand which motifs are important. We used the secondary structure prediction software RNAdraw [53] to analyze the IRES structure of human and mouse origin. In both species, the structure is highly similar and contains three distinct loops (figure 6A). We designed mutations that should result in either loss of IRES function or should be neutral, based on the predicted structure.

IRES*1 and IRES*2 have point mutations in the first and the third stem, respectively, which should, according to RNAdraw, favor a secondary structure very different from the predicted wildtype structure. IRES*3 is predicted to have an even more stable wildtype structure, since a nucleotide introducing a mismatch in the third stem is replaced by a complementary nucleotide. IRES*4 lacks the entire second stem-loop. As expected, the disrupting point mutations almost completely abolished IRES activity, whereas the point mutation strengthening the structure had no significant effect (figure 6B). Surprisingly, deleting the entire second stem-loop didn't completely abolish IRES activity, suggesting the remaining structure is sufficient to give at least partial IRES function.

## Discussion

In this publication we demonstrate the first Polycomb group protein that harbors an IRES in its 5′ UTR, providing a new level of post-transcriptional regulation of a PcG protein complex member. Being only 91 bp in length, the IRES is unusually small yet more active than several other cellular IRESes known to date. Ring1B seems to be largely dependent on the presence of the IRES for its translation, as Ring1B translation is clearly impaired when the IRES is not present. Cryptic promoter activity was excluded by showing lack of expression of downstream ORFs in promoterless constructs. Finally, we show with disrupting and stabilizing point mutations in the IRES sequence that the software-predicted secondary structure is likely to be correct and functional with regard to the IRES activity. Confirming such a regulatory function, the secondary structure is strikingly homologous between mouse and human.

Remarkably, the Ring1B IRES seems to be sensitive to the absence of a translation initiation complex, since it is much less active when the eIF4E binding domain is cleaved off eIF4G by viral protease 2A. This event prevents the formation of an initiation complex and therefore inhibits translational start. Under these conditions, IRES-dependent translation could be the only mechanism for any mRNA to be translated. This observation suggests the Ring1B IRES is to some extend dependent on cap-dependent translational mechanisms. The reason for this could be indirect, in that the Ring1B IRES needs a trans-acting factor that is translated in a cap-dependent manner, but it is currently unclear which factor this could be.

Another possible explanation comes from ribosome shunting, a discontinuous scanning mechanism that in result resembles IRES activity but requires a m^7^guanosine cap. However, shunting requires shunt donor and acceptor sequences to enable scanning ribosomes to bypass mRNA sequences in the 5′ UTR, none of which were found in the Ring1B 5′ UTR. Of note, cap-dependent IRES translation is not unprecedented: the eIF4GI-variant of eIF4G itself was recently found to harbor a cap-dependent IRES, with no clear shunt donor and acceptor sequences [58]. Taken together, this suggests that the IRESes of eIF4G and Ring1B may represent a special subclass of IRES sequences which in part require cap-translation initiation factors for optimal translation.

We speculate that the reason why Ring1B mRNA harbors an IRES, is because Ring1B protein needs to be present in a cell under all circumstances. After testing many primary cell types, tumor cell lines and live tissue, we found no cell type with low levels or lack of Ring1B protein (unpublished observation). All cells tested have readily detectable levels of Ring1B protein. Also, our previous work indicated that Ring1B knockout mice die early in gestation, with severe defects in the mesoderm and deregulated hox gene expression [28], suggesting a vital function for Ring1B in early embryo development. The presence of an IRES might ensure translation of Ring1B in the early embryo under stress conditions such as low oxygen or limited amino acid availability.

Another possible explanation for the need for Ring1B protein to be present at all times comes from differentiation studies with mouse embryonic stem (ES) cells. Recent genome-wide studies show that PcG proteins are critically involved in keeping differentiation-related genes silent in ES cells [3], [4], [6]. Since Ring1B seems to play an essential role in PRC1 and early embryo survival, lack of Ring1B might compromise PRC1 function, leading to unregulated differentiation of stem cells, possibly resulting in cell death. This stem cell based explanation is further supported by the many reports that implicate Bmi1, a Ring1B interaction partner and regulator of Ring1B's ubiquitin E3 ligase activity, in stem cell self-renewal [33]–[36].

The apparent permanent need for Ring1B could be related to its known enzymatic function at the heart of PRC1, acting as a E3 ubiquitin ligase for monoubiquitination of histone H2A. We and others have shown by protein crystallography, that Ring1B and Bmi1 strongly interact via their RING finger domains as well as the Ring1B N-terminal tail, which wraps around Bmi1 [59], [60]. Together these proteins form an active E3 ligase heterodimer, capable of monoubiquitinating H2A more efficiently than Ring1B alone. Truncated versions of both proteins, including the RING finger domains and small adjacent sequences, were shown to be sufficient for heterodimerization, catalytic activity and substrate recognition [59], which leaves more than half of each protein for interaction with other proteins. Since PcG proteins act in large multimeric complexes, it is likely PcG members other than Bmi1 also provide a layer of regulation of Ring1B's E3 ligase activity. This is supported by the finding that an *in vitro* translated PRC1 containing Ring1A, Ring1B, Bmi1 and Pc3 leads to higher levels of uH2A than Ring1B alone [26], suggesting a synergistic effect of PRC1 on H2A monoubiquitination. Importantly, only lack of Ring1B abolishes H2A monoubiquitination [25], indicating PRC1 without Ring1B cannot monoubiquitinate H2A. Indeed, loss of Ring1B results in rapid deubiquitination of H2A and apoptosis [6, Van der Stoop and Boutsma, unpublished observations]. There has been a long-standing correlation between decreased levels of uH2A and apoptosis [61], [62], which might point to the idea that a minimal level of uH2A is necessary for cell survival. This again supports the idea that Ring1B protein needs to be present under all circumstances, a requirement that could possibly be met by the presence of the newly found IRES.

Another possibility is that the IRES in the 5′ UTR increases the stability of the mRNA. This is a common phenomenon to highly structured RNA, both with specific sequences in the 5′ untranslated region as well as in the 3′ untranslated region [63]. Indeed, the Ring1B 3′ UTR can be very large, fragments of up to 3.5 kb were found in the online databases, and its sequence is remarkably conserved between mouse and human, just as its 5′ UTR. Increasing Ring1B mRNA stability in this way could point to a mechanism through which the presence of Ring1B protein is secured by very different means than by maintaining translation, namely by providing a pool of Ring1B mRNA when general transcription is halted.

Taken together, it is very likely that Ring1B protein needs to be present under virtually all circumstances. This idea is now strongly supported by the fact that Ring1B can be translated via its IRES under conditions when traditional cap-dependent translation is impaired, but also by the possibility that the presence of highly structured UTRs in the Ring1B mRNA contribute to its stability and therefore provide a stock of Ring1B mRNA when transcription is impaired. Further experiments on the exact events following the disappearance of Ring1B protein from a cell, preferably via a controlled experimental setup using conditional genetic inactivation, should give more insights into the role of Ring1B in the cell.
